# Single-Molecule Imaging Reveals Dynamics of CREB Transcription Factor Bound to Its Target Sequence

**DOI:** 10.1038/srep10662

**Published:** 2015-06-03

**Authors:** Noriyuki Sugo, Masatoshi Morimatsu, Yoshiyuki Arai, Yoshinori Kousoku, Aya Ohkuni, Taishin Nomura, Toshio Yanagida, Nobuhiko Yamamoto

**Affiliations:** 1 Graduate School of Frontier Biosciences, Osaka University, Osaka, Japan; 2 Graduate School of Engineering Science, Osaka University, Osaka, Japan; 3 Riken Quantitative Biological Center (QBic), Osaka, Japan

## Abstract

Proper spatiotemporal gene expression is achieved by selective DNA binding of transcription factors in the genome. The most intriguing question is how dynamic interactions between transcription factors and their target sites contribute to gene regulation by recruiting the basal transcriptional machinery. Here we demonstrate individual binding and dissociation events of the transcription factor cAMP response element-binding protein (CREB), both *in vitro* and in living cells, using single-molecule imaging. Fluorescent–tagged CREB bound to its target sequence cAMP-response element (CRE) for a remarkably longer period (dissociation rate constant: 0.21 s^-1^) than to an unrelated sequence (2.74 s^-1^). Moreover, CREB resided at restricted positions in the living cell nucleus for a comparable period. These results suggest that CREB stimulates transcription by binding transiently to CRE in the time range of several seconds.

Transcription is the initial step in gene regulation in cells^1^. Assembly and disassembly of the transcription complex, which are governed by protein-DNA and protein-protein interactions, control spatiotemporal transcription within the nuclear space^2^. In particular, specific binding of a transcription factor to its target sequence is a necessary condition for recruitment of basal transcriptional machinery components such as RNA polymerase (RNAP) II and co-regulators^3^. To date, fluorescence recovery after photobleaching (FRAP) and fluorescence correlation spectroscopy (FCS) have demonstrated dynamic behaviour of transcription factors^4^,^5^. However, the kinetics of transcription factor binding to DNA and their spatial distributions in the nucleus remains uncertain.

CREB is a well characterized transcription factor which regulates gene expression in response to cAMP, calcium and cytokine signals in homeostasis and memory formation^6^,^7^. Biochemical and structural analyses have demonstrated that a CREB homodimer binds to a CRE in the promoter regions of its target genes^6^,^8^. Transcription could be initiated by recruitment of the co-activators CREB-binding protein (CBP)/p300 and cAMP-regulated transcriptional co-activators (CRTCs) to CREB via protein-protein interactions in response to the signals^9^,^10^. How do CREB-CRE interactions underlie transcription? How does CREB contribute to selective gene expression by being distributed spatially within the nucleus? Although CREB has been shown to be mobile in living cell nuclei^11^, a key issue is to quantitatively understand the spatiotemporal dynamic behavior of CREB interacting with DNA.

In the present study, we investigated real-time binding and dissociation of fluorescent-tagged CREB to CRE at the single-molecule level *in vitro* and in living cells. Our results show that CREB resides on the CRE sequence for a significantly longer period (more than several seconds) than on an unrelated sequence, but that DNA binding-deficient mutants do not. Dynamic behavior of CREB in living cell nuclei further demonstrates that CREB resides in fixed locations in the time range of seconds. These findings *in vitro* and in living cells support the view that transient and frequent binding of the transcription factor is responsible for gene regulation.

## Results

### Single-Molecule Imaging Reveals Real-Time Interaction between CREB and CRE *in vitro*

**Figure 1:**
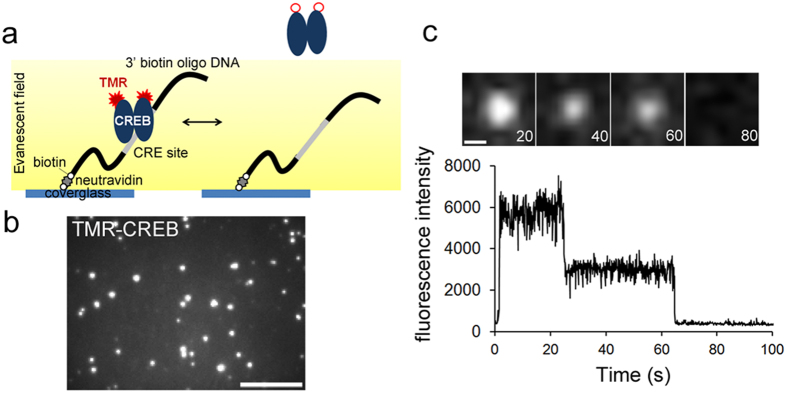
Single-molecule imaging of CREB on a DNA-coated cover glass (**a**) Experimental design of single-molecule imaging to visualize TMR-CREB interacting with surface-tethered DNA using TIRF microscopy. (**b**) TMR-CREB spots interacting with CRE were observed. TIRF images were acquired continuously at 10 frames per second for 120 s. Scale bar: 10 μm. (**c**) TMR-CREB spots exhibit two stepwise reductions of the fluorescence intensity. Scale bar: 0.5 μm.

First, we directly examined interactions between CREB and CRE *in vitro* using single-molecule imaging^12^. To visualize a single molecule of CREB, HaloTag-CREB was labeled with a tetramethylrhodamine (TMR)-conjugated HaloTag ligand (TMR-CREB). We confirmed the ability of HaloTag-CREB to induce downstream transcription (Supplementary Fig. S1). TMR-CREB was loaded into a flow cell comprising two cover glasses coated with oligonucleotides containing the CRE sequence (GCACGTCA) from the human brain-derived neurotrophic factor (*bdnf*) locus^13^ (Fig. 1a). Total internal reflection fluorescence (TIRF) microscopy showed that numerous fluorescent spots of TMR-CREB remained associated with the CRE-coated cover glass for various periods (Fig. 1b and Supplementary Movie S1). Furthermore, some spots clearly exhibited two stepwise reductions in their fluorescence intensity (Fig. 1c), indicating that the CREB dimer interacting with CRE is visualized at the single-molecule level.

### CREB Binds to the CRE Sequence with Long Residence Time

**Figure 2:**
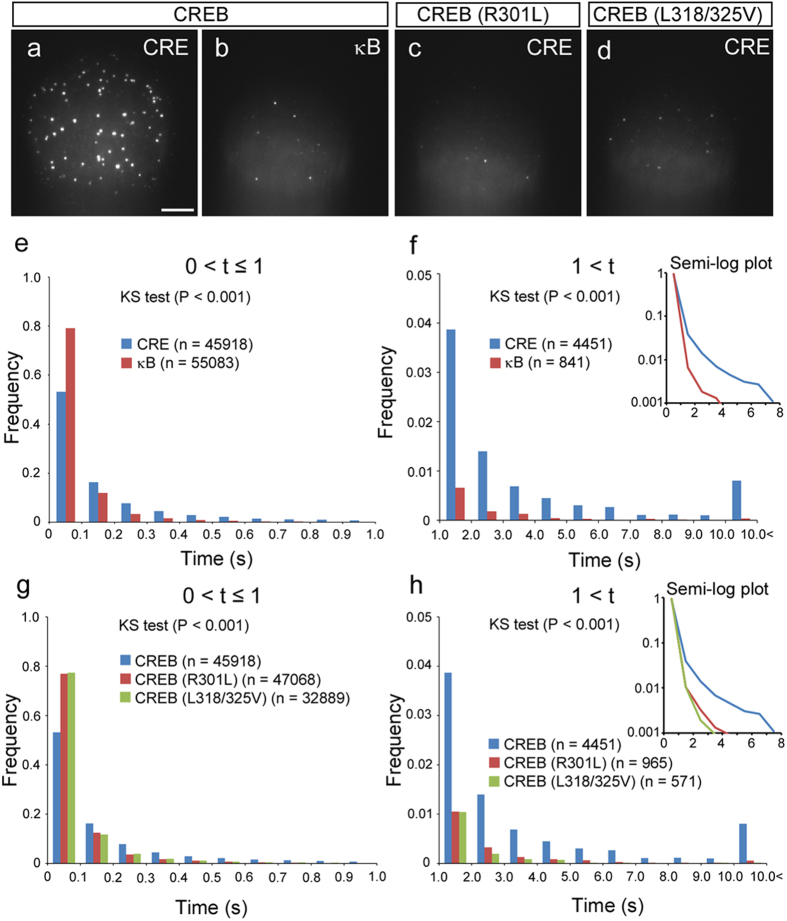
Individual interaction between CREB and CRE *in vitro.* (**a**,**b**) TIRF images show individual TMR-CREB spots interacting with CRE sequence (a; the same image in Fig. 1b) and κB sequence (**b**). Scale bar: 10 μm. (**c**,**d**) TIRF images of TMR-mutant CREB (R301L and L318/325V) interacting with CRE sequence. (**e**,**f**) Quantitative analysis of the residence time of CREB spots interacting with CRE and κB sequences. (**e**) and (**f**) show distribution histograms for short (≤1 s) and long (>1 s) residence times of TMR-CREB, respectively. The number of TMR-CREB spots analyzed is presented in parentheses. The Kolmogorov-Smirnov (KS) test showed that the difference between the two distributions (CRE vs. κB) is significant (P < 0.001). (**g**,**h**) Quantitative analysis of the residence time of mutant CREB spots interacting with CRE sequence. (**g**) and (**h**) show distribution histograms of short (≤1 s) and long (>1 s) residence times of the spots, respectively. The number of TMR-CREB spots analyzed is presented in parentheses. The distribution of wild-type CREB is significantly different from those of the mutants (KS test, P < 0.001).

The residence times of individual CREB dimers bound to the CRE sequence were quantitated. The majority (91.8%) of TMR-CREB spots exhibited short residence times (≤1 s, Fig. 2a,e), whereas a small (8.2%) but significant fraction stayed in the same place for markedly longer periods (>1 s, Fig. 2a,f). In contrast, the fraction of TMR-CREB spots having the longer residence times was substantially lower (2.2%) on a cover glass coated with a recognition sequence for transcription factor NF-κB (the κB site sequence, GGGACTTTCC)^14^, indicating that TMR-CREB has a stronger affinity for the CRE sequence (Fig. 2a,b,e,f, Supplementary Fig. S2a, Supplementary Movie S2).

We also investigated the residence time of mutant CREB (R301L), which is unable to bind to the CRE sequence^15^. As expected, the population with the long residence time (1.9%) was much smaller compared to that for the intact CREB (Fig. 2c,h, Supplementary Fig. S2b, Supplementary Movie S3). Another mutant CREB (L318/325V), which cannot bind to CRE because it cannot dimerize^16^, also displayed a much smaller population with the long residence time (1.5%, Fig. 2d,g,h, Supplementary Fig. S2b and Supplementary Movie S4). This is consistent with previous findings from gel electrophoresis mobility shift assays^15^,^16^. These results indicate that wild-type CREB has sequence-specific binding activity with a long residence time.

The distribution histogram of the residence times of CREB bound to CRE fitted a biexponential function (Supplementary Fig. S2a). The dissociation rate constants for the short and long residences were 2.57 ± 0.33 s^-1^ and 0.21 ± 0.03 s^-1^, respectively. In contrast, the distribution histogram of the residence times of CREB on the κB sequence fitted more accurately a monoexponential function with a dissociation rate constant of 2.74 ± 0.12 s^-1^, which is close to the short residence in the CREB-CRE interaction (Supplementary Fig. S2a).

To test the possibility that photobleaching of TMR-CREB might have affected the rate constant, we examined the fluorescence stability of TMR-CREB in the same condition. The time constant of the photobleaching (13.8 s) was much longer than the duration of the long-residence interaction between CREB and CRE sequence (1 / 0.21 = 4.8 s) (Fig. S2a, c). Thus, it is likely that the residence times of CREB on the CRE sequence-coated substratum reflect its binding to CRE.

### Single-Molecule CREB in Living Cell Nuclei

**Figure 3:**
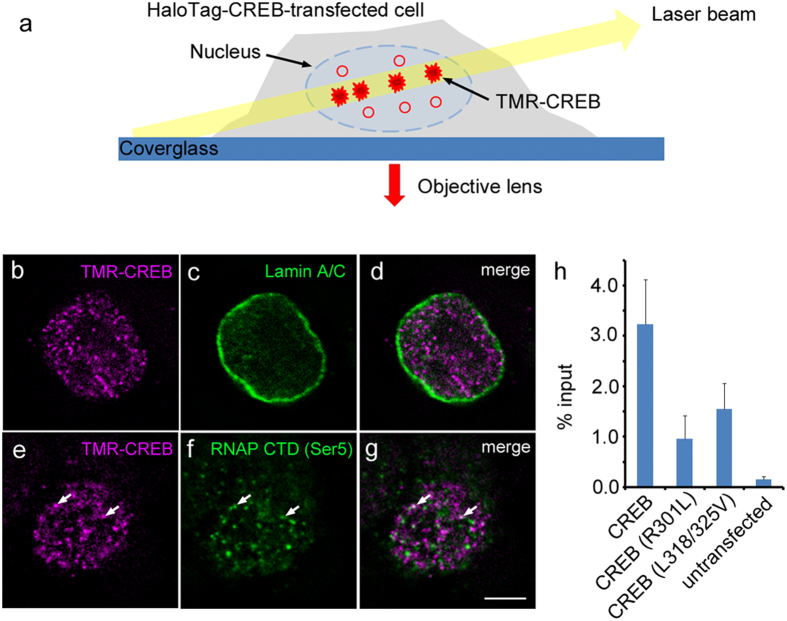
Single-molecule imaging of CREB in the nucleus. (**a**) Experimental design of single-molecule imaging to visualize TMR-CREB in the nucleus of living cells. A limited number of TMR-CREB molecules in the nucleus are observed with low background using HILO microscopy. (**b**–**g**) Subcellular localization of TMR-CREB in fixed HaloTag-CREB-transfected Neuro2a cells using HILO fluorescence microscopy. Scale bar: 10 μm. Immunocytochemical staining was performed with anti-lamin A/C antibody (**c**) anti-Ser5-phosphorylated RNAP II C-terminal domain antibody (**f**) and TMR-conjugated HaloTag ligand (**b**,**e**) Series of images (0.2 μm × 5 sections) were subjected to a deconvolution algorithm. Arrows indicate TMR-CREB spots colocalized with Ser5-phosphorylated RNAP II C-terminal domain. (**h**) ChIP and qPCR were performed to detect HaloTag-CREB residing at the CRE sequence within the *c-fos* promoter in cells transfected with plasmids encoding wild-type, mutant (R301L) or mutant (L318/325V) HaloTag-CREBs. Bar represents the mean ± SEM from three independent experiments.

We next studied the *in vivo* dynamics of CREB in the nuclei of living mammalian cells at the single-molecule level^17^. Mouse neuroblastoma Neuro2a cells were transfected with HaloTag-CREB. To observe single-molecule TMR-CREB in the nucleus, highly inclined and laminated optical sheet (HILO) microscopy was used^18^ (Fig. 3a). Moreover, HaloTag-CREB was sparsely labeled by adding a low concentration of TMR-HaloTag ligand, in order to follow the dynamics of individual TMR-CREB molecules.

Before live imaging, immunocytochemical analysis was performed with HILO microscopy to examine the subcellular localization of TMR-CREB in the transfected cells. A few dozen fluorescent spots of TMR-CREB were clearly observed, with low background signals (Fig. 3b,e). In addition, TMR-CREB was predominantly localized within the nucleus, surrounded by lamin A/C, a nuclear envelope component^19^ (Fig. 3b–d). Moreover, some of the TMR-CREB spots were found to colocalize with Ser5-phosphorylated RNAP II C-terminal domain, which suggests that a proportion of TMR-CREB molecules are present at actively transcribed loci^20^ (Fig. 3e–g). The average number of colocalizing spots was 2.4 ± 1.4 per optical section (n = 40 cells) (Supplementary Fig. S3).

To confirm that TMR-CREB was binding to endogenous CRE sequences in transfected cells, we performed chromatin immunoprecipitation (ChIP) assays with HaloTag affinity resin (Fig. 3h). Wild-type CREB was associated with the promoter region of *c-fos* as well as *bdnf*, each of which contains a CRE sequence^8^,^13^. In contrast, the binding fraction of both the mutant CREB (R301L) and the mutant CREB (L318/325V) apparently decreased in accordance with the observation *in vitro* (Fig. 2g,h).

### *In vivo* Dynamics of CREB in the Nucleus of Living Cells

**Figure 4:**
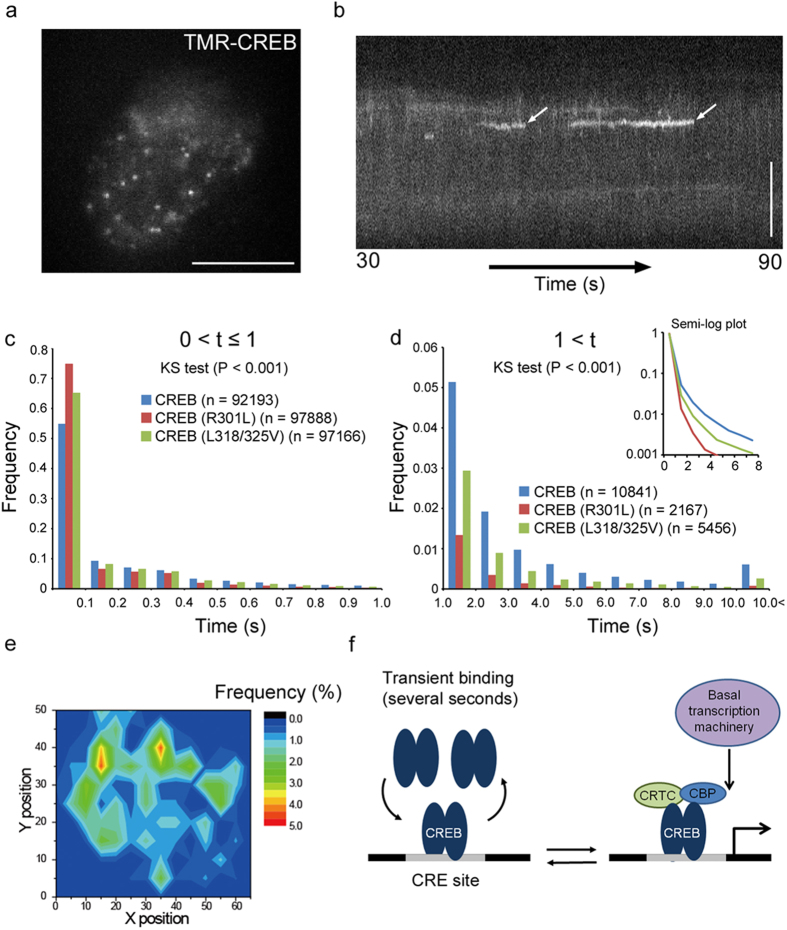
Dynamics of CREB in the nucleus of living cells. (**a**) TMR-CREB spots were observed in the living cell nucleus by HILO fluorescence microscopy. Average of the images (for 1 s) shows individual TMR-CREB spots in the nucleus. Scale bar: 10 μm. (**b**) A representative kymogram (60 s) shows the long residence times of TMR-CREB spots at restricted locations (arrows). Scale bar (y-axis): 10 μm. (**c**,**d**) Distribution histograms show the short (≤1 s) and long (>1 s) residence times, respectively, of TMR-CREB and TMR-mutant CREBs in living cells. The number of spots analyzed in living cells is presented in parentheses. The distribution of wild-type CREB is significantly different from those of mutants (KS test, P < 0.001). (**e**) A color contour map represents spatial distribution of the frequency of appearance of TMR-CREB spots having long residence times (>1 s) in a living cell. The area covering the whole nucleus was divided into 154 equal sub-areas (1.2 × 1.2 μm). The number of TMR-CREB spots for the entire observation time (120 s) was counted in each sub-area. Twelve out of a total of 270 spots (4.4%) accumulated in two different sub-areas (hot points, orange/red). This accumulation level for the hot points is much higher than would be expected (1.8 spots, 0.7%) in a random distribution. TMR-CREB spots also tended to occur frequently in the sub-areas surrounding the hot points. (**f**) A model of the dynamic interaction of CREB with CRE to promote transcription initiation.

Real-time movements of TMR-CREB in living cells were investigated by HILO microscopy, and were compared to those of mutant CREB (R301L and L318/325V). If the above *in vitro* observations apply to the interactions of CREB with genomic DNA, TMR-CREB spots having the longer residence times (several seconds) should be detected more frequently than those of TMR-mutant CREB. Many TMR-CREB spots were clearly visible in the nuclei of living cells, with a wide range of residence times (Fig. 4a and Supplementary Movie S5) which were quantified as described above. Although the majority of both CREB (89.5%) and mutant CREB (R301L: 97.8%, L318/325V: 94.6%) spots disappeared by 1 s, the fraction with longer residence times (>1 s) was larger for wild-type CREB (10.5%) than for the mutant CREBs (R301L: 2.2%, L318/325V: 5.4%) (Fig. 4c,d, Supplementary Fig. S4, and see also Supplementary Fig. S5). The differences between wild-type and the mutant CREBs in living cells were smaller than those *in vitro*, suggesting that CREB may interact with nuclear proteins in addition to DNA in the nucleus. Moreover, the dissociation rate constants for TMR-CREB (3.11 ± 0.53 s^-1^ and 0.35 ± 0.08 s^-1^ for the short and long residence components, respectively) in living cells were not only different from those for the mutant CREBs but also comparable with those *in vitro* (Supplementary Fig. S2b, S4, Supplementary Table S1). Taken together with the findings in ChIP assay (Fig 3h), these results suggest that the fraction with the long residence time most likely corresponds to CREB specific binding to CRE. The fraction of short residence time indicates the non-specific binging to DNA. Indeed, we observed the substantial fraction of binding remained even in the mutant CREB transfected cells in ChIP assays. The subtle difference between the mutant CREB (R301L) and the mutant CREB (L318/325V) in ChIP assay also seems to reflect the residence time.

The spatial distribution of TMR-CREB spots in the nucleus was also studied. We found that TMR-CREB spots with long residence times (>1 s) appeared repeatedly at highly restricted locations in the nucleus (Fig. 4b). A location at which >4% of the long-residence-time TMR-CREB spots accumulated was defined as a hot point. As exemplified in Fig. 4e, TMR-CREB spots accumulated in two different hot points. The average number of hot points per optical section was 3.0 ± 0.6 (n = 22 cells), similar to the number of active RNAP II foci which colocalized with TMR-CREB spots (2.4 ± 1.4 per optical sections; Fig. 3g, Supplementary Fig. S3). Taken together, these results suggest that TMR-CREB interacts with genomic DNA, on presumed multiple CRE sites, in living cells, with a similar time course to that *in vitro*.

## Discussion

The present study has shown, for the first time, a dynamic interaction between CREB and CRE at the single-molecule level. While previous DNase I footprinting and gel-shift assays had indicated a strong affinity between the two^6^, our findings both *in vitro* and in living cells revealed the existence of two modes of interaction, with a short and a long residence time. The interaction of CREB with CRE is therefore more dynamic than previously suggested^21^,^22^,^23^.

The short residence may represent CREB interacting weakly with non-specific DNA sequences while it searches for CRE sites in the genome. Consistent with this view, non-specific DNA binding by the *lac* repressor (LacI) shows very fast dissociation in living *Escherichia coli* cells^24^. Several recent studies have also demonstrated that mammalian transcription factors very transiently interact with chromatin for less than 1 s^25^,^26^,^27^,^28^,^29^,^30^ (Supplementary Fig. S5), although FRAP analysis of CREB in PC12 cells exhibits slow dissociation^11^. On the other hand, the long residence of CREB may reflect the specific interaction with CRE, since the long-residence fraction was almost abolished when CREB was incubated with the κB sequence (Fig. 2f). Similar residence times were obtained in living cells by single-molecule imaging (Fig. 4c,d). Therefore, we propose that CREB participates in transcription initiation by binding to the target sequence transiently in the time range of several seconds (Fig. 4f). In accordance with this view, recent single-molecule imaging studies have demonstrated that other mammalian transcription factors transiently reside in the nucleus with a similar time course of second order^25^,^26^,^27^,^28^,^29^,^30^. In particular, p53 and the glucocorticoid receptor exhibit the long residence at the target sites^27^.

The presence of hot points in the nucleus further implies that CREB repeatedly binds to and dissociates from active CRE sites^31^,^32^,^33^. CBP/p300 and CRTCs may bind efficiently only to CREB dimers residing on these CRE sites. Previous studies with tandem gene arrays have also demonstrated that transcription factors bind repeatedly at the active gene loci^34^,^35^,^36^,^37^. Thus, it is likely that the repetitive binding of transcription factors promotes transcription^38^,^39^.

Our spatial analysis also showed that a few TMR-CREB hot points were present in a single focal plane, in accordance with the number of TMR-CREB spots which colocalized with Ser5-phosphorylated RNAPII foci in fixed cells (Fig. 3g,4e, Supplementary Fig. S3). One may suppose that this number of the hot points was fewer than the total number of CRE sites in the whole genome^31^,^32^. However, it is not surprising because only a fraction of CRE sites is active in living cells^33^,^40^,^41^,^42^,^43^. As chromatin states and chromosome positions in the interphase nucleus are known to influence gene expression^4^,^44^, the formation of the hot point is probably limited by these chromatin components. Therefore, although further study is needed, the hot points may indicate active gene loci whose transcription is driven by the CREB-CRE interaction.

Collectively, our observations indicate that CREB contributes to transcription initiation by binding transiently to genomic CRE sites in the time range of several seconds. In addition, the present analysis using single-molecule imaging both *in vitro* and in living cells will enable further studies of the dynamics of transcription factors on DNA.

## Methods

### Plasmids

pFN21AB5414 containing HaloTag-human CREB1 cDNA was purchased from Promega. To generate the HaloTag-human CREB1 R301L and L318/325V mutant, point mutations were introduced into pFN21AB5414 by PCR-mediated site-directed mutagenesis with the mutagenic primer pairs: R301L 5’-GAAGCAGCTCGAGAGTGTCCTAGAAAGAAGAAAGAATATG-3' and its complementary oligonucleotide; L318/325V 5’-TTTAGAAAACAGAGTGGCAGTGGTTGAAAATCAAAACAAGAC AGTGATTGAGGAGCTA-3' and its complementary oligonucleotide.

### DNA Coating on Cover Glasses

Cover glasses (Matsunami) were cleaned by boiling in RCA solution (6:4:1 H_2_O/30% NH_4_OH/30% H_2_O_2_) for 1 h. Poly(ethyleneimine) (PEI) and poly(acrylic acid) (PAcr) (Sigma) were dissolved at 2 mg/ml in H_2_O and then adjusted to pH 8.0 using either NaOH or HCl. The cleaned cover glasses were immersed in positive (+, PEI) and negative (−, PAcr) polyelectrolytes in the sequence + / wash / - / wash / + / wash for 30 min each at room temperature; each wash step involved three rinses in distilled water^45^. A flow cell was assembled from two cover glasses and two strips of double-sided tape as spacers. The flow cell was treated with 10 mg/ml biotinylated α-casein in PBS for 5 min and then reacted with 5 mg/ml NeutrAvidin (Life Technologies) in PBS. Biotin-labeled dsDNAs (40 nM in PBS) were adsorbed onto the cover glass surface through the avidin-biotin interaction for 5 min. Biotin-labeled 19-nt dsDNA including the CRE sequence was prepared by annealing 5’-GACAGCGCACGTCAAGGCA-biotin-3’ and its complementary oligonucleotide. Biotin-labeled 22-nt dsDNA including the κB sequence was prepared by annealing 5’-AGTTGAGGGGACTTTCCCAGGC-biotin-3’ and its complementary oligonucleotide. After each procedure, the flow cell was washed with PBS. Finally, the flow cell was treated with 10 mg/ml BSA in PBS for 5 min and then flushed with a binding buffer solution (100 mM KCl, 1 mM MgCl_2_, 20 mM HEPES-NaOH (pH 7.8), 0.1 M DTT, 2 mg/ml BSA, 50% sucrose). In experiments, TMR-CREB or TMR-mutant CREB in binding buffer was loaded into the flow cell. All CREB proteins were generated by the TNT Quick Coupled Transcription/Translation System (Promega) and labeled with HaloTag TMR Ligand (Promega). To determine fluorescence stability, TMR-CREB in binding buffer was loaded into a 35-mm glass-bottom culture dish (Greiner bio-one) without DNA coating.

### Cell Culture

Mouse Neuro2a cells were cultured in DMEM/F12 medium (Life Technologies) supplemented with 10% fetal bovine serum (Hyclone). The cells were seeded to 80% confluence in a 4-well culture plate (Nalge Nunc) and then transfected with plasmid vectors. All plasmids used for transfection were obtained using PureLink Hipure Plasmid DNA purification kits (Life Technologies) and dissolved in PBS. Transfection with Lipofectamine2000 (Life Technologies) was performed according to the manufacturer’s instructions. After transfection, cells were cultured for 8 h and then replated on a 35-mm glass-bottom culture dish (Greiner bio-one). To label HaloTag CREB with a TMR, HaloTag CREB cDNA-transfected cells were incubated with growth medium containing 0.1 nM HaloTag TMR Ligand (Promega) for 15 min in a CO_2_ incubator, after which the medium was replaced.

### Microscopy

An inverted microscope (Ti-E, Nikon) with oil-immersion objective (100 x, NA 1.49; Nikon) was used for all experiments. Alexa 488 and TMR were excited by 488- (20 mW; Coherent) and 561-nm (20 mW; Coherent) lasers, respectively. TIRF and HILO were used as illumination for *in vitro* and *in vivo* analysis, respectively. All fluorescence live images were obtained at 10 fps using an EM-CCD (iXon897, Andor Technology) with Solis software (Andor Technology). The images shown in the Figures are averaged representations of photos captured over 1 or 5 s. To observe the dynamics of TMR-CREB in living cells, the cell culture dish was mounted on a stage top incubator (Tokai Hit) maintained at 37 °C in an environment of humidified 5% CO_2_, 20% O_2_, and 75% N_2_. All images were analyzed by ImageJ software with a self-made plugin (Particle Track and Analysis). Fluorescence images of fixed cells were acquired and processed with NIS Element software linked to a deconvolution module (Nikon).

### Statistical Analysis

All statistical values are presented as the mean value ± SD from at least three independent experiments. Significant differences were determined with Student’s *t*-test or the Kolmogorov-Smirnov test. Origin (OriginLab) and Excel (Microsoft) were used for statistical analysis and data plotting.

### Immunostaining

Neuro2a cells were fixed at room temperature for 10 min in 4% paraformaldehyde/PBS. They were then permeabilized and blocked for 10 min in buffer G, composed of 5% normal goat serum (S-1000; Vector Labs) and 0.1% Triton X-100 in PBS. The cells were then incubated overnight at 4 °C with anti-lamin A/C mouse monoclonal antibody (3A6-4C11; Active Motif) at 1:1000 and anti-RNAP II C-terminal domain phospho Ser5 rabbit polyclonal antibody (Active Motif) at 1:10000 in buffer G. Primary antibodies were detected by incubation with Alexa 488-conjugated anti-mouse IgG (A11029; Invitrogen) at 1:400, or with Alexa 488-conjugated anti-rabbit IgG (A-11034; Invitrogen) at 1:400, in buffer G at room temperature for 2 h. Nuclei were stained with 0.1% 4’,6-diamidino-2-phenylindole (Sigma) in a mounting medium containing 50% glycerol and 2.3% 1,4-diazabicyclo[2.2.2]octane (Sigma) in 50 mM Tris-HCl (pH 8.0).

### ChIP Assay and Quantitative real-time PCR (qPCR)

Cells were cross-linked with 1% formaldehyde in culture medium for 10 min, and lysed in lysis buffer (50 mM Tris-HCl (pH 7.5), 150 mM NaCl, 1% Triton X-100, 0.1% sodium deoxycholate). Cross-linked cell lysates were sonicated for 20 cycles (burst and intervals of 15 s each) with a VCX130PB (Amplitude 60%; Sonics & Materials). Sonicated samples were incubated with HaloLink Resin (Promega) for 3 h at room temperature. The resin was washed twice with nuclease-free water, once with high-salt buffer (50 mM Tris-HCl (pH 7.5), 700 mM NaCl, 1% Triton X-100, 0.1% sodium deoxycholate and 1 mM EDTA), and finally three times with nuclease-free water. Samples were reverse cross-linked with reversal buffer (10 mM Tris-HCl (pH 8.0), 1 mM EDTA and 300 mM NaCl) for 6 h at 65 °C. The eluted DNA was purified using a PCR product purification kit (Promega). qPCR was performed using an ABI StepOne (Applied Biosystems). To quantify the amplified DNA, a SYBR Green PCR assay was used with KAPA SYBR FAST qPCR Kit Master Mix (KAPA Biosystems) and the primers 5'-GCGTAGAGTTGACGACAGAGC-3’ and 5'-TGGATGGACTTCCTACGTCA-3’. The obtained values were normalized to the respective input DNA.

## Additional Information

**How to cite this article**: Sugo, N. *et al.* Single-Molecule Imaging Reveals Dynamics of CREB Transcription Factor Bound to Its Target Sequence. *Sci. Rep.* **5**, 10662; doi: 10.1038/srep10662 (2015).

## Supplementary Material

Supplementary Information

Supplementary Movie S1

Supplementary Movie S2

Supplementary Movie S3

Supplementary Movie S4

Supplementary Movie S5

## References

[b1] Maniatis T. & Reed R. An extensive network of coupling among gene expression machines. Nature 416, 499–506 (2002). 11932736 10.1038/416499a

[b2] Ptashne M. & Gann A. Transcriptional activation by recruitment. Nature 386, 569–577 (1997). 9121580 10.1038/386569a0

[b3] Kadonaga J. T. Regulation of RNA polymerase II transcription by sequence-specific DNA binding factors. Cell 116, 247–257 (2004). 14744435 10.1016/s0092-8674(03)01078-x

[b4] Hager G. L., McNally J. G. & Misteli T. Transcription dynamics. Mol. Cell 35, 741–753 (2009). 19782025 10.1016/j.molcel.2009.09.005 PMC6326382

[b5] Michelman-Ribeiro A. *et al.* Direct measurement of association and dissociation rates of DNA binding in live cells by fluorescence correlation spectroscopy. Biophys. J. 97, 337–346 (2009). 19580772 10.1016/j.bpj.2009.04.027 PMC2711375

[b6] Mayr B. & Montminy M. Transcriptional regulation by the phosphorylation-dependent factor CREB. Nat. Rev. Mol. Cell Biol. 2, 599–609 (2001). 11483993 10.1038/35085068

[b7] Lonze B. E. & Ginty D. D. Function and regulation of CREB family transcription factors in the nervous system. Neuron 35, 605–623 (2002). 12194863 10.1016/s0896-6273(02)00828-0

[b8] Roesler W. J., Vandenbark G. R. & Hanson R. W. Cyclic AMP and the induction of eukaryotic gene transcription. J. Biol. Chem. 263, 9063–9066 (1988). 2837474

[b9] Conkright M. D. *et al.* TORCs: transducers of regulated CREB activity. Mol Cell 12, 413–423 (2003). 14536081 10.1016/j.molcel.2003.08.013

[b10] Chrivia J. C. *et al.* Phosphorylated CREB binds specifically to the nuclear protein CBP. Nature 365, 855–859 (1993). 8413673 10.1038/365855a0

[b11] Mayr B. M., Guzman E. & Montminy M. Glutamine rich and basic region/leucine zipper (bZIP) domains stabilize cAMP-response element-binding protein (CREB) binding to chromatin. J. Biol. Chem. 280, 15103–15110 (2005). 15703171 10.1074/jbc.M414144200

[b12] Harada Y. *et al.* Single-molecule imaging of RNA polymerase-DNA interactions in real time. Biophys. J. 76, 709–715 (1999). 9929475 10.1016/S0006-3495(99)77237-1 PMC1300075

[b13] Fang H. *et al.* Transcriptional activation of the human brain-derived neurotrophic factor gene promoter III by dopamine signaling in NT2/N neurons. J. Biol. Chem. 278, 26401–26409 (2003). 12738784 10.1074/jbc.M211539200

[b14] Lenardo M.J. & Baltimore D. NF-kappa B: a pleiotropic mediator of inducible and tissue-specific gene control. Cell 58, 227–229 (1989). 2665943 10.1016/0092-8674(89)90833-7

[b15] Walton K. M., Rehfuss R. P., Chrivia J. C., Lochner J. E. & Goodman R. H. A dominant repressor of cyclic adenosine 3',5'-monophosphate (cAMP)-regulated enhancer-binding protein activity inhibits the cAMP-mediated induction of the somatostatin promoter *in vivo*. Mol. Endocrinol. 6, 647–655 (1992). 1350057 10.1210/mend.6.4.1350057

[b16] Dwarki V. J., Montminy M. & Verma I. M. Both the basic region and the 'leucine zipper' domain of the cyclic AMP response element binding (CREB) protein are essential for transcriptional activation. EMBO J. 9, 225–232 (1990). 2136830 10.1002/j.1460-2075.1990.tb08099.x PMC551651

[b17] Sako Y. & Yanagida T. Single-molecule visualization in cell biology. Nat. Rev. Mol. Cell Biol. **Suppl**, SS1–5 (2003). 14587519

[b18] Tokunaga M., Imamoto N. & Sakata-Sogawa K. Highly inclined thin illumination enables clear single-molecule imaging in cells. Nat. Methods 5, 159–161 (2008). 18176568 10.1038/nmeth1171

[b19] Gerace L., Blum A. & Blobel G. Immunocytochemical localization of the major polypeptides of the nuclear pore complex-lamina fraction. Interphase and mitotic distribution. J. Cell Biol. 79, 546–566 (1978). 102651 10.1083/jcb.79.2.546 PMC2110258

[b20] Phatnani H. P. & Greenleaf A. L. Phosphorylation and functions of the RNA polymerase II CTD. Genes Dev. 20, 2922–2936 (2006). 17079683 10.1101/gad.1477006

[b21] Benbrook D. M. & Jones N. C. Different binding specificities and transactivation of variant CRE's by CREB complexes. Nucleic Acids Res 22, 1463–1469 (1994). 8190638 10.1093/nar/22.8.1463 PMC308006

[b22] Bullock B. P. & Habener J. F. Phosphorylation of the cAMP response element binding protein CREB by cAMP-dependent protein kinase A and glycogen synthase kinase-3 alters DNA-binding affinity, conformation, and increases net charge. Biochemistry 37, 3795–3809 (1998). 9521699 10.1021/bi970982t

[b23] Euskirchen G. *et al.* CREB binds to multiple loci on human chromosome 22. Mol Cell Biol 24, 3804–3814 (2004). 15082775 10.1128/MCB.24.9.3804-3814.2004 PMC387762

[b24] Elf J., Li G. W. & Xie X. S. Probing transcription factor dynamics at the single-molecule level in a living cell. Science 316, 1191–1194 (2007). 17525339 10.1126/science.1141967 PMC2853898

[b25] Groeneweg F.L. *et al.* Quantitation of Glucocorticoid Receptor DNA-Binding Dynamics by Single-Molecule Microscopy and FRAP. PLoS One 9, e90532 (2014). 24632838 10.1371/journal.pone.0090532 PMC3954550

[b26] Mazza D., Abernathy A., Golob N., Morisaki T. & McNally J. G. A benchmark for chromatin binding measurements in live cells. Nucleic Acids Res. 40, e119 (2012). 22844090 10.1093/nar/gks701 PMC3424588

[b27] Morisaki T., Müller W. G., Golob N., Mazza D. & McNally J. G. Single-molecule analysis of transcription factor binding at transcription sites in live cells. Nat. Commun. 5, 4456 (2014). 25034201 10.1038/ncomms5456 PMC4144071

[b28] Gebhardt J. C. *et al.* Single-molecule imaging of transcription factor binding to DNA in live mammalian cells. Nat. Methods 10, 421–426 (2013). 23524394 10.1038/nmeth.2411 PMC3664538

[b29] Speil J. *et al.* Activated STAT1 transcription factors conduct distinct saltatory movements in the cell nucleus. Biophys. J. 101, 2592–2600 (2011). 22261046 10.1016/j.bpj.2011.10.006 PMC3297778

[b30] Chen J. *et al.* Single-molecule dynamics of enhanceosome assembly in embryonic stem cells. Cell 156, 1274–1285 (2014). 24630727 10.1016/j.cell.2014.01.062 PMC4040518

[b31] Impey S. *et al.* Defining the CREB regulon: a genome-wide analysis of transcription factor regulatory regions. Cell 119, 1041–1054 (2004). 15620361 10.1016/j.cell.2004.10.032

[b32] Zhang X. *et al.* Genome-wide analysis of cAMP-response element binding protein occupancy, phosphorylation, and target gene activation in human tissues. Proc. Natl. Acad. Sci. USA 102, 4459–4464 (2005). 15753290 10.1073/pnas.0501076102 PMC555478

[b33] Cha-Molstad H., Keller D. M., Yochum G. S., Impey S. & Goodman R. H. Cell-type-specific binding of the transcription factor CREB to the cAMP-response element. Proc. Natl. Acad. Sci. USA 101, 13572–13577 (2004). 15342915 10.1073/pnas.0405587101 PMC518796

[b34] McNally J. G., Müller W. G., Walker D., Wolford R. & Hager G. L. The glucocorticoid receptor: rapid exchange with regulatory sites in living cells. Science 287, 1262–1265 (2000). 10678832 10.1126/science.287.5456.1262

[b35] Bosisio D. *et al.* A hyper-dynamic equilibrium between promoter-bound and nucleoplasmic dimers controls NF-kappaB-dependent gene activity. EMBO J. 25, 798–810 (2006). 16467852 10.1038/sj.emboj.7600977 PMC1383558

[b36] Rayasam G. V. *et al.* Ligand-specific dynamics of the progesterone receptor in living cells and during chromatin remodeling *in vitro*. Mol. Cell Biol. 25, 2406–2418 (2005). 15743833 10.1128/MCB.25.6.2406-2418.2005 PMC1061598

[b37] Sharp Z. D. *et al.* Estrogen-receptor-alpha exchange and chromatin dynamics are ligand- and domain-dependent. J. Cell Sci. 119, 4101–4116 (2006). 16968748 10.1242/jcs.03161

[b38] Cisse I. I. *et al.* Real-time dynamics of RNA polymerase II clustering in live human cells. Science 341, 664–667 (2013). 23828889 10.1126/science.1239053

[b39] Stasevich T. J. *et al.* Regulation of RNA polymerase II activation by histone acetylation in single living cells. Nature 516, 272–275 (2014). 25252976 10.1038/nature13714

[b40] Conkright M. D. *et al.* Genome-wide analysis of CREB target genes reveals a core promoter requirement for cAMP responsiveness. Mol. Cell 11, 1101–1108 (2003). 12718894 10.1016/s1097-2765(03)00134-5

[b41] Iannello R. C. *et al.* Methylation-dependent silencing of the testis-specific Pdha-2 basal promoter occurs through selective targeting of an activating transcription factor/cAMP-responsive element-binding site. J. Biol. Chem. 275, 19603–19608 (2000). 10766751 10.1074/jbc.M001867200

[b42] Martinowich K. *et al.* DNA methylation-related chromatin remodeling in activity-dependent BDNF gene regulation. Science 302, 890–893 (2003). 14593184 10.1126/science.1090842

[b43] Iguchi-Ariga S. M. & Schaffner W. CpG methylation of the cAMP-responsive enhancer/promoter sequence TGACGTCA abolishes specific factor binding as well as transcriptional activation. Genes Dev. 3, 612–619 (1989). 2545524 10.1101/gad.3.5.612

[b44] Lanctôt C., Cheutin T., Cremer M., Cavalli G. & Cremer T. Dynamic genome architecture in the nuclear space: regulation of gene expression in three dimensions. Nat. Rev. Genet. 8, 104–115 (2007). 17230197 10.1038/nrg2041

[b45] Kartalov E. P., Unger M. A. & Quake S. R. Polyelectrolyte surface interface for single-molecule fluorescence studies of DNA polymerase. Biotechniques 34, 505–510 (2003). 12661156 10.2144/03343st02

