# Supplementary material for: Single-Molecule Imaging Reveals Dynamics of CREB Transcription Factor Bound to Its Target Sequence
Source: Sci Rep. 2015 Jun 3;5:10662. doi: 10.1038/srep10662 (PMC4454023; doi:10.1038/srep10662)
Supplement: Supplementary Information [file srep10662-s1.pdf]

Supplementary Information

Single-Molecule Imaging Reveals Dynamics of CREB Transcription Factor Bound to Its Target Sequence

Noriyuki Sugo, Masatoshi Morimatsu, Yoshiyuki Arai, Yoshinori Kousoku, Aya Ohkuni, Taishin Nomura, Toshio Yanagida & Nobuhiko Yamamoto

Table S1. TMR-CREB dissociation rate constants in Neruo2a cells

	total number of observed cells	dissociation rate constant (s^{-1}) (mean ± S.D.)	
		short residence	long residence
CREB	36	3.11 ± 0.53	0.35 ± 0.08
CREB (R301L)	21	4.25 ± 0.67	0.52 ± 0.15
CREB (L318/325V)	31	3.77 ± 0.78	0.43 ± 0.13

Supplementary Figure S1

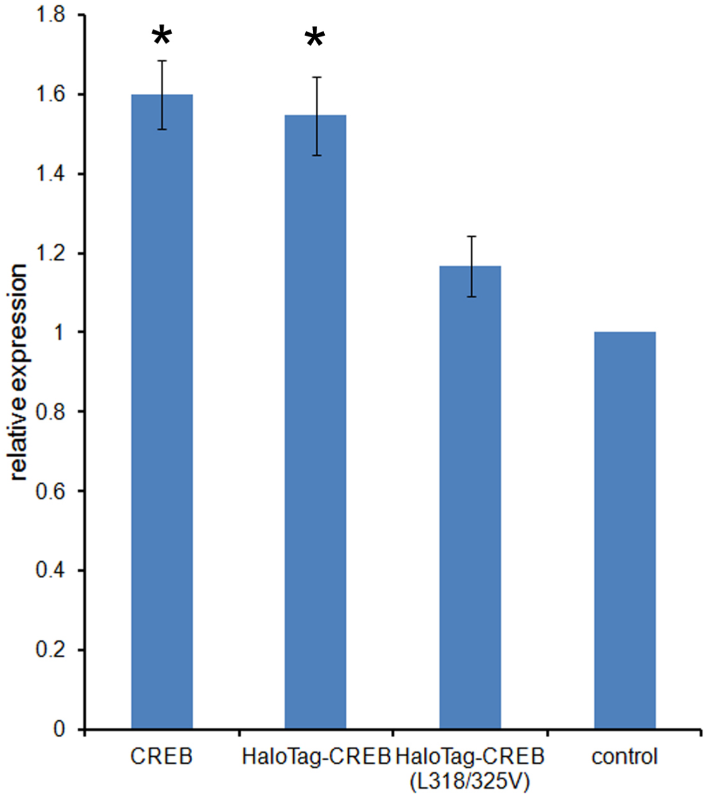

Figure S1. Ability of tagged CREB proteins as a transcription factor. To confirm the ability of HaloTag-CREB to induce transcription, we examined the expression of endogenous *c-fos*, a CREB target gene, in HaloTag-CREB transfected cells. The expression level of *c-fos* mRNA was quantified in CREB-, HaloTag-CREB- and HaloTag-CREB (L318/325V)-transfected Neuro2a cells, and compared to that in non-transfected control cells, using qRT-PCR. HaloTag-CREB increased endogenous *c-fos* expression to the same level as did wild-type CREB, whereas mutant CREB did not increase it significantly. Bars represent the mean ± SEM from three independent experiments. Asterisks indicate a significant difference from non-transfected control cells (Student's *t*-test; *P < 0.01).

Supplementary Figure S2

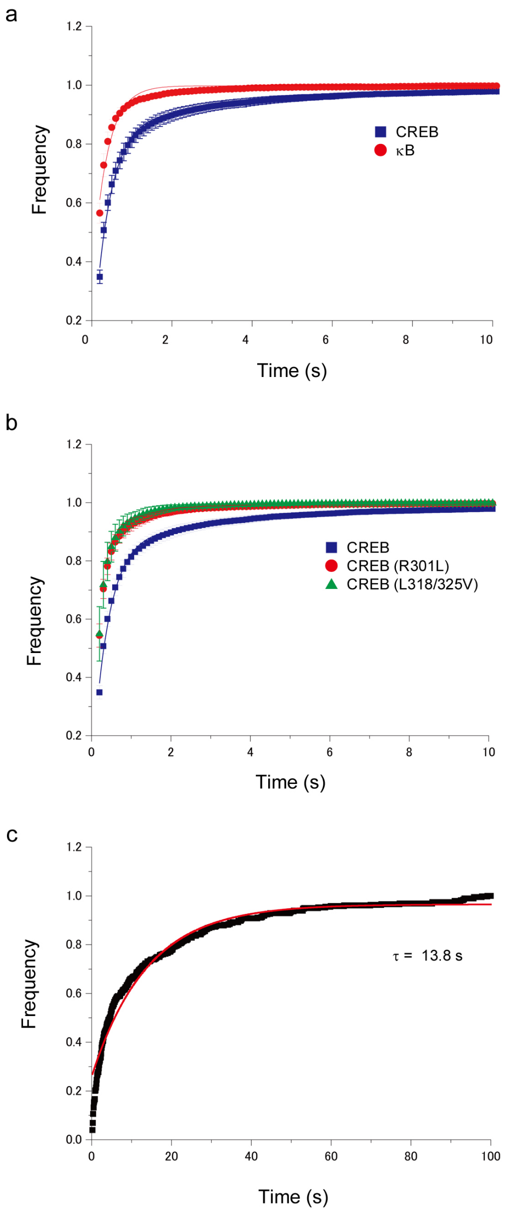

Figure S2. Quantitative analysis of residence time of CREB spots *in vitro*. (**a**) Cumulative plots of the residence time of TMR-CREB bound to CRE (blue) and κB (red). Bars represent the mean ± SD from nine independent observations. The curves fitted mono- (κB: red line) or biexponential (CRE: blue line) functions. (**b**) Cumulative plots of the residence times of TMR wild-type CREB and TMR mutant CREBs (R301L and L318/325V) on the CRE sequence. Bars represent the mean ± SD from nine independent observations. (**c**) Cumulative probability distribution of the photobleaching time of TMR-CREB (n = 379 spots). The result fitted a mono-exponential function (red line).

Supplementary Figure S3

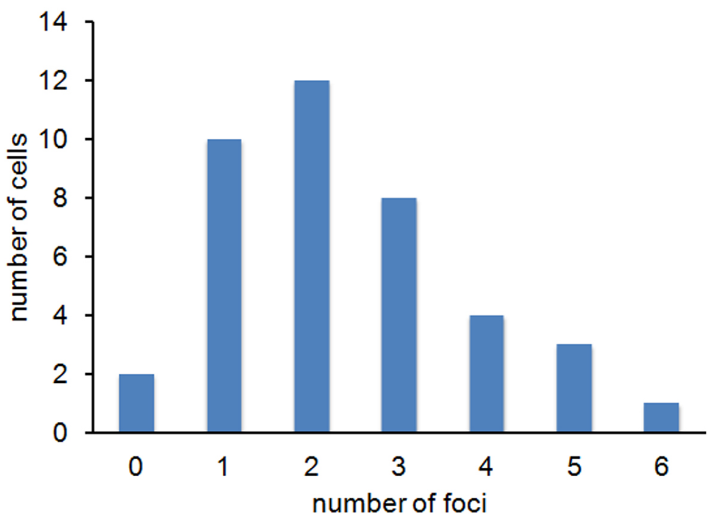

Figure S3. The number of colocalized spots of TMR-CREB and Ser5-phosphorylated RNAP II C-terminal domain. The positions of both types of spot were determined in five consecutive 0.2-µm optical sections in fixed cells (n = 40 cells). The average number of colocalized spots was 2.4 ± 1.4.

Supplementary Figure S4

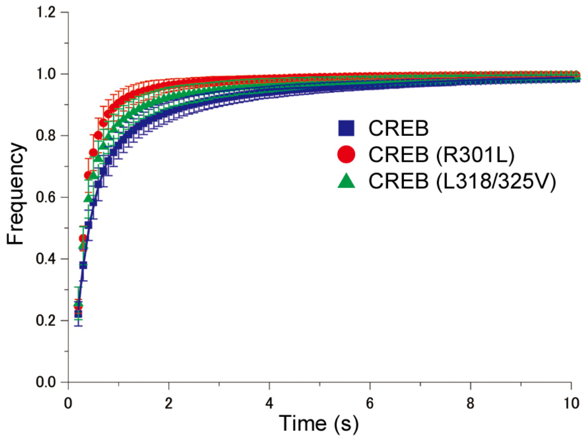

Figure S4. Quantitative analysis of residence time of CREB spots in living cells. Cumulative plots of the residence times of wild-type CREB (n = 36 cells) and mutant CREBs (n =21 cells for R301L, n = 31 cells for L318/325V) in living cells. Bars represent the mean ± SD from independent observations. The result for wild-type CREB fitted a biexponential function (blue line).

Supplementary Figure S5

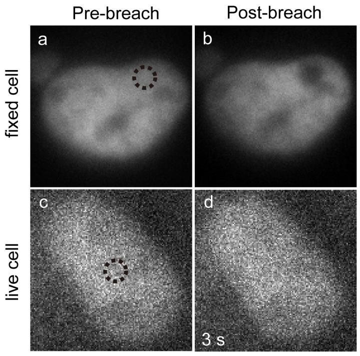

Figure S5. FRAP analysis of TMR-CREB in Neuro2a cells. (**a, b**) Irradiation of 561nm laser (max power, 15 s) efficiently generated a clear dark spot in paraformaldehyde-fixed TMR-CREB expressing Neuro2a cells. A dotted circle indicates the position of irradiation. (**c, d**) A dark spot in living cells shortly (3 s) after the same irradiation could not be detected. A dotted circle indicates the position of irradiation. The total nuclear TMR-CREB fluorescent intensities were reduced slightly after irradiation. Thus, it is likely that most TMR-CERB molecules move very rapidly. This is consistent with our observation that 89.5 % of TMR-CREB spots resided in the same place only for less than 1 s. In accordance with this view, FRAP analysis in HaloTag-p53–transfected cells show a similar image to that in our observation (Mazza, et al., 2012).

Supplementary Movie Legends

Movie S1. Individual interaction between CREB and CRE sequence *in vitro.*

Imaging of TMR-CREB interacting with CRE sequence. Movie speed is real time.

Movie S2. Individual interaction between CREB and κB sequence *in vitro.*

Imaging of TMR-CREB interacting with κB sequence. Movie speed is real time.

Movie S3. Individual interaction between mutant CREB (R301L) and CRE sequence *in vitro.*

Imaging of TMR-mutant CREB (R301L) interacting with CRE sequence. Movie speed is real time.

Movie S4. Individual interaction between CREB (L318/325V) and CRE sequence *in vitro.*

Imaging of TMR-mutant CREB (L318/325V) interacting with CRE sequence. Movie speed is real time.

Movie S5. Dynamics of CREB in the nucleus of living cells.

Imaging of TMR-CREB in a HaloTag-CREB-transfected living cell nucleus. Movie speed is real time.

Supplementary Methods

qRT-PCR

Total RNA was purified with Total RNA Extraction Miniprep System (VIOGENE). Reverse transcription was carried out using Transcriptor First Strand cDNA Synthesis Kit (Roche Diagnostics) according to the manufacturer's instructions. To examine *c-fos* expression profiles in Neuro2a cells, quantitative real time PCR was performed using an ABI StepOne (Applied Biosystems). TaqMan Gene Expression Assay (Assay ID Mm00487425_m1; Applied Biosystems) and Universal ProbeLibrary Mouse GAPD Gene Assay (Roche Applied Science), respectively, were performed to quantify the expression levels of *c-fos* and the housekeeping gene *gapdh* as an endogenous control. Data were analyzed by the comparative cycle threshold ($\Delta\Delta C_T$) method. The analysis was normalized to expression of *gapdh*.

FRAP analysis

An inverted epifluorescence microscope (Ti-E, Nikon) with oil-immersion objective (100 x, NA 1.49; Nikon) was used for all experiments. To breach a spot in TMR-CREB expressing cells, 15 s irradiation of 561 nm laser (20 mW; Coherent) was carried out through a pinhole for paraformaldehyde-fixed or living cells. To observe the dynamics of TMR-CREB in living cells, the cell culture dish was mounted on a stage top incubator (Tokai Hit) maintained at 37°C in an environment of humidified 5% CO_2, 20% O_2, and 75% N_2. All fluorescence images were obtained at 1 fps using an EM-CCD (iXon897, Andor Technology) with NIS Element software (Nikon).
